# Epitranscriptome insights into *Riccia fluitans* L. (Marchantiophyta) aquatic transition using nanopore direct RNA sequencing

**DOI:** 10.1186/s12870-024-05114-4

**Published:** 2024-05-15

**Authors:** Mateusz Maździarz, Katarzyna Krawczyk, Mateusz Kurzyński, Łukasz Paukszto, Joanna Szablińska-Piernik, Monika Szczecińska, Paweł Sulima, Jakub Sawicki

**Affiliations:** 1 Department of Botany and Evolutionary Ecology, University of Warmia and Mazury in Olsztyn, Plac Łódzki 1, Olsztyn, 10-719 Poland; 2 Department of Genetics, Plant Breeding and Bioresource Engineering, University of Warmia and Mazury in Olsztyn, Plac Łódzki 3, Olsztyn, 10-724 Poland

**Keywords:** *Riccia fluitans*, Gene expression, Transcript expression, Methylation, Poliadenylation, Non-adenine residue, Environmental adaptation

## Abstract

**Background:**

*Riccia fluitans*, an amphibious liverwort, exhibits a fascinating adaptation mechanism to transition between terrestrial and aquatic environments. Utilizing nanopore direct RNA sequencing, we try to capture the complex epitranscriptomic changes undergone in response to land-water transition.

**Results:**

A significant finding is the identification of 45 differentially expressed genes (DEGs), with a split of 33 downregulated in terrestrial forms and 12 upregulated in aquatic forms, indicating a robust transcriptional response to environmental changes. Analysis of N6-methyladenosine (m6A) modifications revealed 173 m6A sites in aquatic and only 27 sites in the terrestrial forms, indicating a significant increase in methylation in the former, which could facilitate rapid adaptation to changing environments. The aquatic form showed a global elongation bias in poly(A) tails, which is associated with increased mRNA stability and efficient translation, enhancing the plant’s resilience to water stress. Significant differences in polyadenylation signals were observed between the two forms, with nine transcripts showing notable changes in tail length, suggesting an adaptive mechanism to modulate mRNA stability and translational efficiency in response to environmental conditions. This differential methylation and polyadenylation underline a sophisticated layer of post-transcriptional regulation, enabling *Riccia fluitans* to fine-tune gene expression in response to its living conditions.

**Conclusions:**

These insights into transcriptome dynamics offer a deeper understanding of plant adaptation strategies at the molecular level, contributing to the broader knowledge of plant biology and evolution. These findings underscore the sophisticated post-transcriptional regulatory strategies *Riccia fluitans* employs to navigate the challenges of aquatic versus terrestrial living, highlighting the plant’s dynamic adaptation to environmental stresses and its utility as a model for studying adaptation mechanisms in amphibious plants.

**Supplementary Information:**

The online version contains supplementary material available at 10.1186/s12870-024-05114-4.

## Background

Direct native RNA sequencing is a novel method for sequencing RNA molecules in their native form without needing to first reverse transcribe them into cDNA. This is made possible by Oxford Nanopore Technologies’ nanopore sequencers which can directly sequence native RNA strands as they pass through a protein nanopore [1, 2]. Unlike traditional sequencing methods, direct RNA sequencing can identify RNA modifications, which are typically erased by widely used sequencing-by-synthesis (SBS) methods [3]. This method has been used to document nucleotide modifications and 3′ polyadenosine tails on RNA strands without added chemistry steps [4]. Direct RNA sequencing allows for the analysis of native RNA strands without reverse transcription or amplification, avoiding biases introduced by these steps (Vacca et al. 2022, Soneson et al. 2019).

Over the past few years, direct RNA sequencing accuracy and throughput have improved to the point that it can offer valuable biological insights. For example, it has revealed capping patterns in human mRNAs [5], detected novel pseudouridine sites in yeast [6], and quantified changing modification levels under stress [7]. As the technology continues advancing, direct sequencing of full-length native RNA strands promises to transform transcriptomics.

Direct RNA sequencing has some limitations to consider. Current protocols require high-quality input RNA, with recommendations of at least 50ng of intact mRNA for optimal throughput [8]. This high RNA input requirement could pose challenges for studies with limited biological material [9]. Additionally, the protocols rely on the poly(A) tail for adapter ligation, restricting the analysis to polyadenylated transcripts and limiting the characterization of non-polyadenylated RNAs [10]. The throughput of direct RNA sequencing is also currently lower than short-read methods based on cDNA sequencing. This can restrict the depth of characterization possible for complex transcriptomes [11]. Finally, computational tools tailored for analyzing the direct sequencing data are still in early development, making data analysis more difficult than established pipelines for short-read data [12]. Further advances in methods and tools will help address these current limitations of direct RNA sequencing, including increased output and error reduction in incoming RNA004 kits.

While direct RNA sequencing has some limitations, it holds particular promise for studying non-model organisms exhibiting remarkable environmental adaptability. These organisms, like amphibious plants that adjust their morphology and physiology to thrive in fluctuating environments. Recent advances in genomics and transcriptomics have shed light on the genetic mechanisms underlying aquatic adaptation. Comparative transcriptomics of amphibious plants grown submerged versus on land reveal differentially expressed genes involved in underwater acclimation like cuticle and stomatal development, cell elongation, and modified photosynthesis [8]. Genomics has also uncovered key roles of plant hormones in regulating heterophylly [13]. Moreover, comparative genomics between aquatic and terrestrial species identify genomic signatures enabling adaptation to submerged life, including changes in submergence tolerance, light sensing, and carbon assimilation genes [14]. However, genomic resources for amphibious plants remain scarce especially in the non-vascular evolutionary lineage.

*Riccia fluitans* is an aquatic liverwort that serves as an excellent model for studying amphibious plants. As one of the earliest diverging land plants, liverworts represent a critical transition point between aquatic and terrestrial environments [15]. *R. fluitans* possess remarkable adaptability, growing floating mats in water or moist soil [15, 16]. When submerged, *R. fluitans* adopts a specialized water form with thin thalli to maximize surface area for gas exchange. Within days of emerging from the water, it can completely alter its morphology into a land form with thicker thallus that reduces water loss. It also stockpiles starch preparing for periodic drought [16, 17]. This extreme plasticity enables the exploitation of both aquatic and terrestrial realms. Its ability to dynamically transform morphology and physiology demonstrates exceptional environmental responsiveness. Understanding the molecular mechanisms behind adaptation to aquatic environments in amphibious plants has been an area of active research. Recent advances in genomics and transcriptomics have enabled new insights into these processes [18]. Genomics provides an overview of the complete genome content of an organism and allows comparisons between related species. Transcriptomics analyzes genome-wide gene expression patterns in different conditions like submergence. Together, these powerful approaches have shed light on the genetic basis of aquatic adaptation. Several studies have compared transcriptomes of amphibious plants grown in aquatic and terrestrial conditions. These analyses have identified differentially expressed genes and pathways involved in underwater adaptation such as cuticle development, stomatal patterning, cell elongation, and photosynthesis. Other studies have also uncovered the roles of plant hormones like abscisic acid, ethylene, and gibberellic acid in regulating heterophylly. Additionally, comparative genomics between aquatic and terrestrial plant species has revealed genomic signatures of adaptation to aquatic life, like changes in genes related to submergence tolerance, light sensing, and carbon assimilation. However, genomic resources for amphibious plants are still limited. Expanding genomic and transcriptomic data from diverse amphibious species and their terrestrial relatives will provide further insights into the evolution of aquatic adaptation in plants. Liverworts are a group of non-vascular plants that are thought to be some of the earliest land plants, evolving around 470 million years ago [19]. Some liverwort species are amphibious, meaning they can thrive in both aquatic and terrestrial environments. One such species is the aquatic liverwort *Riccia fluitans*, which can grow floating mats in water as well as on moist land. Moreover, as a popular aquarium plant that species can be grown fully submerged, forming dense green mats and carpets along the substrate and hardscapes. When growing in water, *R. fluitans* develop thin stems and finely divided leaves that increase surface area for gas exchange. On land, it can alter its morphology within days, developing thicker stems and larger, entire leaves that reduce water loss. It also accumulates starch reserves in preparation for periods of drought [16]. *R. fluitans* is able to switch from vegetative to reproductive growth based on environmental conditions, ensuring reproduction occurs at the right time [15]. The ability to undergo rapid morphological changes allows amphibious liverworts to take advantage of both aquatic and terrestrial habitats. Elucidating the adaptations underlying such plasticity provides perspective on water-to-land transitions of early land plants over 400 million years ago [15, 16]. As an amphibious plant that flourishes both submerged and on moist land, *R. fluitans* serves as a prime model for examining adaptive mechanisms to alternating hydrological regimes. The recent establishment of genetic transformation methods unlocks additional potential for exploring the genetic basis of aquatic acclimation in this liverwort [15].

In this study, we analyze land and water forms of *Riccia fluitans* using nanopore native RNA sequencing technology to verify if this technology could provide additional insight into short-read characterized transcriptomes as well as potential epitranscriptomics changes during adaptation to aquatic environments, which wasn’t studied in liverworts so far.In this study, we delve into the exploration of land and water forms of *R. fluitans* through the application of nanopore native RNA sequencing technology. Our primary focus is to ascertain whether this advanced technology can offer supplementary insights into transcriptomes previously characterized using short-read sequencing. Additionally, we seek to investigate potential epitranscriptomics alterations that occur during the adaptation of liverworts to aquatic environments, a topic that has hitherto remained unexplored in liverworts.

## Results

### Native RNA unveils additional DEGs and DETs compared to cDNA

Sequencing procedures produced 2 × 580,290,571 and 9,238,584 short- and long reads, respectively. The eight sequencing libraries for both technologies distributed 72,536,321 and 1,154,823 mean raw reads per library. After trimming short raw reads, 2 × 514,565,651 sequences survived the quality checkpoint (Additional file 2: Table S1).

**Fig. 1:**
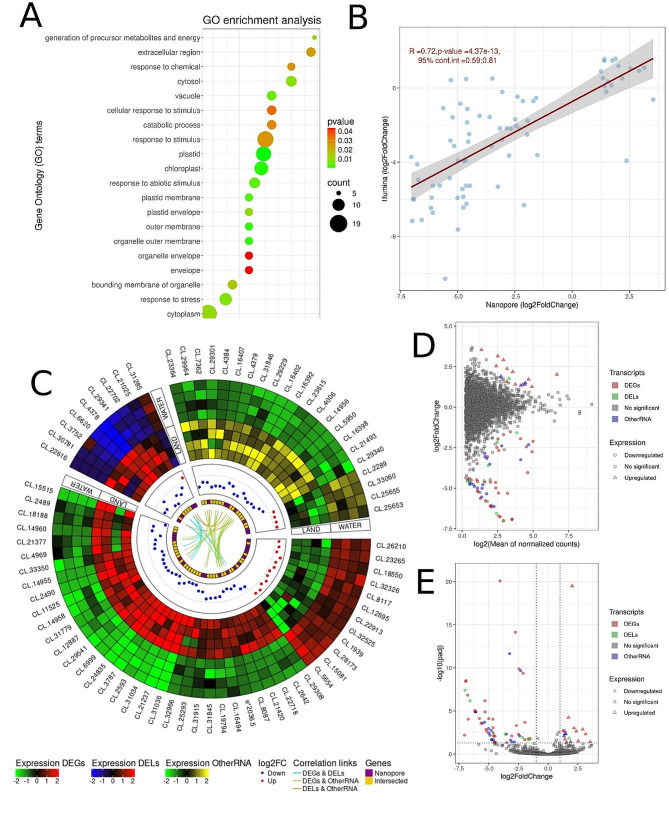
Gene expression profiling of land and water form of *Riccia fluitans* based on direct RNA. **A.** Dotplot chart of enrichment ontology of genes. The circles represent pathways described along the y-axis, colors reflect the adjusted *p*-value of enrichment statistics, and the sizes of the circles represent the number of genes enriched in each pathway. **B.** The dotplot illustrates the correlations between statistically significant genes from nanopore sequencing and genes from Illumina sequencing. The x-axis represents log2FoldChange values for nanopore sequencing, while the y-axis depicts log2FoldChange values for Illumina sequencing. The red line highlights the Pearson correlations. The R value, *p*-value and confidence interval (conf.int) are displayed in the upper left corner. The gray bands around the line represent the standard error of the regression line. **C.** Circular plot depicting the relationships between significant genes. The first track shows 3 heatmaps, which show the expression level in each of the significant genes. The green-black-red scale represents the expression of DEGs, the blue-black-red scale represents the expression of DELs, and the green-black-yellow scale represents the expression of other RNAs. The second track describes the log2FoldChange values of upregulated (red) and downregulated (blue) genes. The third track shows the unique genes that were found in the nanopore data analysis (purple color) as well as the common genes (gold color) in the differential analysis in Illumina and Nanopore. The internal track depicts the correlation relationships, where the blue link represents the correlations between DEGs and DELs, green for DEGs and other RNAs, and orange for DELs and other RNA. **D.** The MA plot visualizes the association between log2FoldChange and log2 from the average of normalized counts. The x-axis displays the log2 of the average of the normalized gene counts, while the y-axis illustrates the log2FoldChange for the gene. Squares represent irrelevant transcripts, triangles represent upregulated transcripts, circles represent downregulated transcripts, and the green color indicates DELs, red indicates DEGs, blue indicates other RNAs, and grey indicates no significant genes. **E.** Volcano plot depicting log2 Fold Change (log2FC) for significant genes. The x-axis displays the log2FC values for each gene, while the y-axis shows the negative log-adjusted *p*-value (p-adjusted). The horizontal dashed line represents the negative logarithmic p-adjusted cutoff value (0.05), and the two vertical lines equal the absolute value of 1 log2FC. Coloured points indicate statistically significant genes, while grey points represent non-significant genes

Using direct RNA sequencing, the genes were characterized according to coding potential to 12,051 expressed active regions, of which 8,043 were classified as protein coding, 1,326 as long non-coding RNAs, and 2,677 were classified as other RNAs. DE analysis provided information about 76 significant genes between land and water *Riccia* form. The 45 genes were signed as differentially expressed genes (DEGs), of which 33 were downregulated (land-specific) and 12 were upregulated (water-specific). The logarithmic value of fold change (log2FC) for DEGs ranged from − 7.02 to 3.54. Deep transcriptome analysis revealed 9 differentially expressed long non-coding RNAs (DELs) (8 down- and 1 upregulated) under land-water environmental change. The log2FC values for DELs were in the range from − 6.98 to 1.76. Additionally, the differential analysis revealed 18 land-specific (with the lowest log2FC = -6.13) and four water-specific (with the highest log2FC = 1.89) expression fluctuations for other RNA (Additional file 2: Table S2). Co-expression analysis revealed 8 trans-interactions between DEGs - DELs, 25 *trans*-interactions between DEG and other RNA, and 4 *trans*-interactions between DELs - and other RNAs. All interactions were positively correlated based on the Pearson coefficient (Additional file 2: Table S3). The expression profiles of all DEGs, DELs, and other RNAs were presented in a volcano plot (Fig. 1D) MA-plot (Fig. 1E) and heatmap enriched by *trans-*interactions (Fig. 1C). All significant 76 genes were checked by Illumina RNA-seq results (Additional file 2: Table S4). The correlation across the expression modification (obtained by Illumina and Nanopore) for these genes was calculated and the coefficient showed a high value equal to 0.72. (Fig. 1B). Interesting that one DEG - evm.TU.utg2036_2952540_3002010__.5 (annotated as Chlorophyll A-B binding family protein) was expressed in Nanopore direct RNA only in plants grown under terrestrial conditions, but has no transcription in any group sequenced by Illumina technology. The log2FC of 6 significant genes (with Gene ID; CL.12,695, CL.21,377, CL.25,655, CL.29,541, CL.31,779, CL.32,326) from direct RNA sequencing did not overlap with the signature of genes from Illumina sequencing. Ontology analysis revealed significance for 192 functional processes which included cytoplasm (GO:0005737; 19 genes), response to stimulus (GO:0050896; 16), plastid (GO:0009536; 14), chloroplast (GO:0009507; 12), response to stress (GO:0006950; 10), and response to abiotic stimulus (GO:0009628; 8.) (Additional file 2: Table S5 and Fig. 1A).

**Fig. 2:**
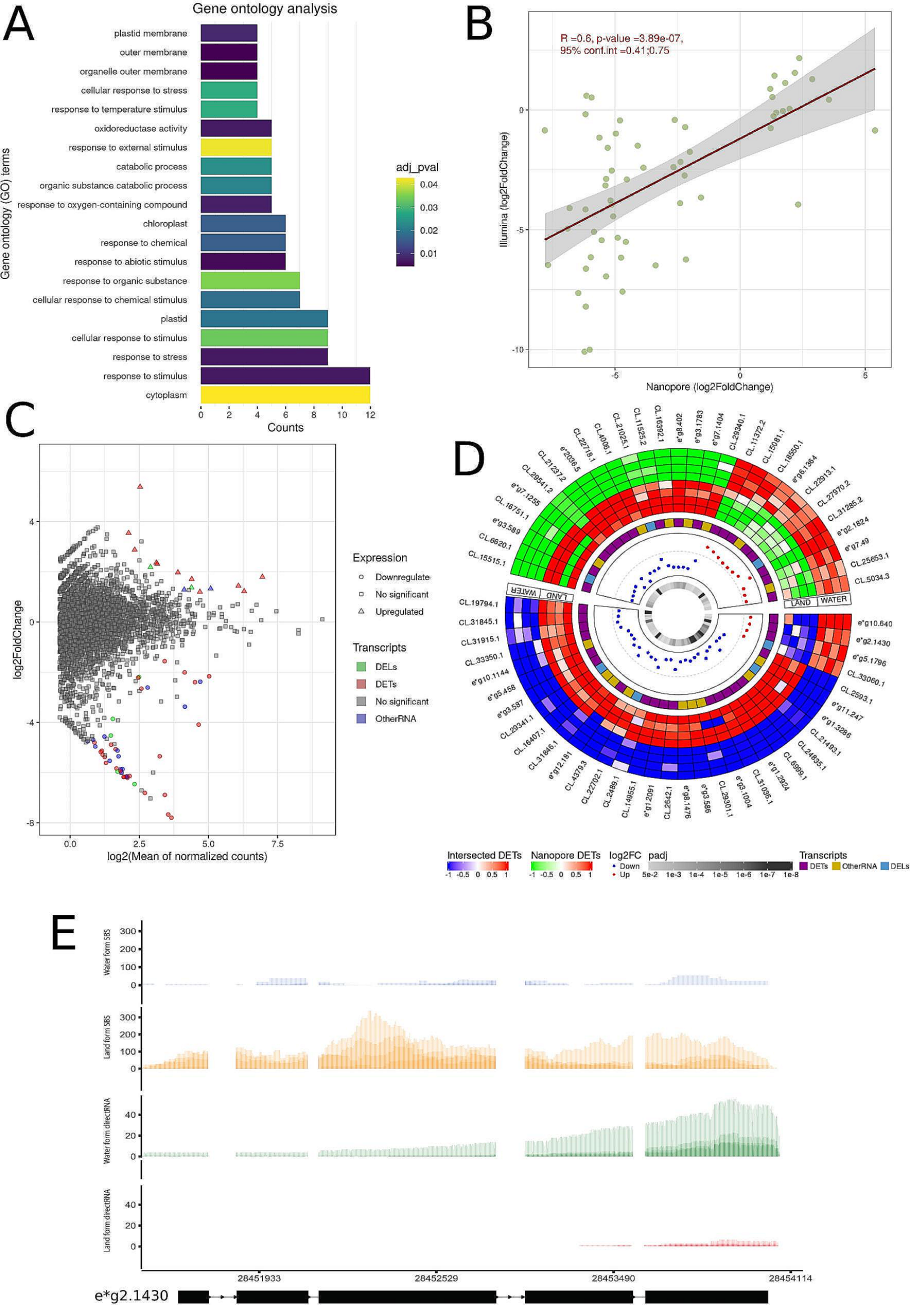
Transcript expression differentiation between land and water form of *Riccia fluitans*. **A.** The barplot depicts the GO annotation distribution for genes exhibiting statistically significant expression differences. The 20 most enriched GO terms were presented. The bars represent the number of genes involved in a particular process. The colors correspond to different adjusted p-values. **B.** The dotplot illustrates the correlations between statistically significant transcripts from nanopore sequencing and transcripts from Illumina sequencing. The x-axis represents log2FoldChange values for nanopore sequencing, while the y-axis depicts log2FoldChange values for Illumina sequencing. The red line highlights the Pearson correlations. The R value, p-value and confidence interval (conf.int) is displayed in the upper left corner. The gray bands around the line represent the standard error of the regression line. **C.** The MA plot visualizes the association between log2FoldChange and log2 from the average of normalized counts. The x-axis displays the log2 of the average of the normalized transcript counts, while the y-axis illustrates the log2FoldChange for the transcript. Squares represent irrelevant transcripts, triangles represent upregulated transcripts, circles represent downregulated transcripts, and the green color indicates DELs, red indicates DETs, blue indicates other RNA, and gray indicates no significant transcripts. **D.** A circular chart depicting two circular heatmaps representing the expression levels of significant genes in eight samples encompassing four terrestrial and four aquatic forms of *R. fluitans*. The heatmaps correspond to Illumina-intersected DETs (Intersected DETs) and unique DETs to Nanopore (Nanopore DETs). The green-white-red color scale represents Nanopore DETs, while the blue-white-red color scale signifies intersected DETs. The subsequent track exhibits a heatmap showcasing purple for DETs, gold for other RNA, and light blue for DELs. The third track illustrates the differential expression values (log2FoldChange) between upregulated (red) and downregulated (blue) genes in each comparison group. The inner heatmap depicts the p-value adjusted for each gene. **E.** Incongruent results of SBS and direct RNA DEG analysis. Gene evm.model.group2.1430 of unknown function (containing fasciclin-like domain) is downregulated in water form based on SBS RNA-seq analysis and upregulated in nanopore direct RNA sequencing

Information on the expression of specific transcripts was also revealed by direct RNA. An analysis of transcript expression showed similar results, while differences in *Riccia fluitans* response to environmental changes were found to be significant and more detailed. The transcript level analyses revealed expression of 17,064 mRNAs in both land and aquatic form of *R. fluitans*. The 61 transcripts were classified as significant, of which 46 transcripts increased expression in land condition and 15 had higher expression in aquatic condition. The distribution of log2FC values ranged from − 7.8 to 5.39. Among DETs, 38 were identified as protein coding, while 7 and 16 were classified as DELs and other RNAs (Additional file 2: Table S6). The distributions of DETs, DELs, and OtherRNA were presented in a MA-plot (Fig. 2C and Additional file 1: Fig. S1) and a circular plot with a heatmap (Fig. 2D). The direct RNAs expression values for DETs, DELs and other RNAs were correlated with Illumina sequencing data. In the result the Pearson coefficient was equal to 0.6 (Fig. 2B). Among DELs, two transcripts with unknown function (CL.16,392,CL.16,402; CL.16392.1 and evm.model.group3.1783) exhibited expression solely in *R. fluitans* grown under land conditions, while Illumina sequencing failed to detect any expression for both transcripts. Interestingly, our results revealed the eight transcripts with opposite expression trends in the use of Nanopore and Illumina sequencing. The most divergent expression profile detection showed transcript (CL.12,695; evm.model.group2.1430) with largest log2FC (form − 3.95 to 2.32) fluctuations in Illumina and Nanopore, respectively (Fig. 2E and Additional file 2: Table S6 and S7). The transcripts were annotated to the 201 GO terms (FDR < 0.05), such as response to stimulus (GO:0050896), cytoplasm (GO:0005737), response to stress (GO:0006950), cellular response to stimulus (GO:0051716), and plastid (GO:0009536) (Fig. 2A and Additional file 2: Table S8). Native RNA revealed 27 additional statistically significant genes (Fig. 1D and E and Additional file 2: Table S2, Table 4 and Additional file 1: Fig. S2 and Fig. S3) and 28 statistically significant transcripts (Fig. 2C and Additional file 2: Table S6 and Table S7 and Additional file 1: Fig. S1, Fig. S4 and Fig. S5) through gene and transcript differential analysis, respectively, when compared to cDNA.

### Water environmental increases RNA methylation

**Fig. 3:**
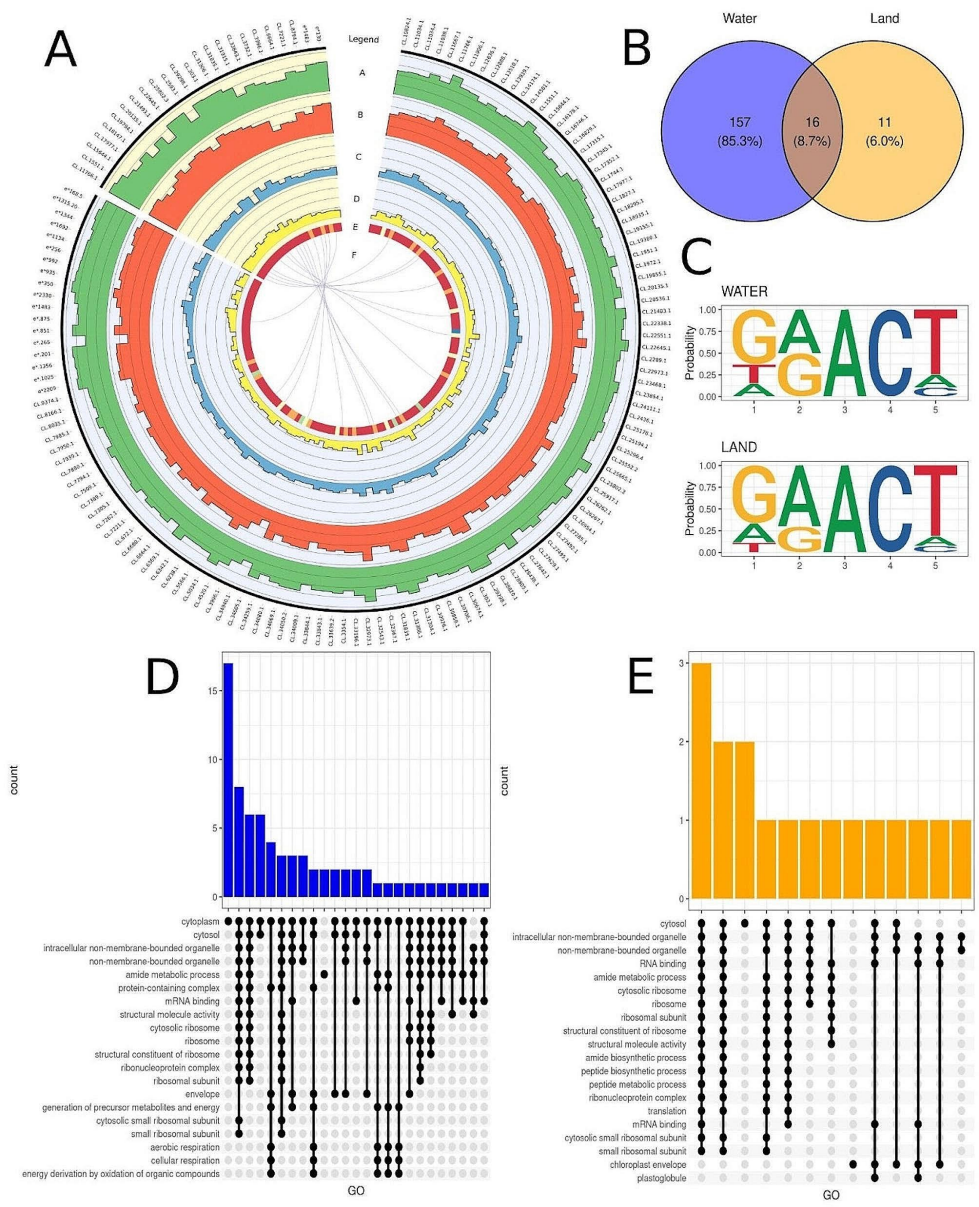
Methylation signature of land and water form of *Riccia fluitans*. **A.** The histogram tracks (**A**-**D**) depict the logarithmic mean of expression patterns from the Illumina (**A**, **B**) and Nanopore (**C**, **D**) sequencing of the frequency water (**A**, **C**) and land (**B**, **D**) forms of *R. fluitans*. The heatmap (**E**) represents the methylation levels of the transcript, with red indicating one methylation, orange indicating two methylations, yellow indicating three methylations, green indicating four, and blue indicating five methylations. The innermost track (**F**) presents the correlation links between experimental groups, where purple links depict intersected transcripts with methylations. **B.** The Venn diagram depicts the number and percentage of unique methylations in terrestrial (orange), aquatic (blue), and common to both sets (dark orange) *R. fluitans*. **C.** A logo diagram depicts the probability of a nucleotide appearing in the first five positions of the significant methylation motif in both the water (upper diagram) and land (lower diagram) forms. The larger the letter representing the nucleotide, the higher the probability of its appearance. **D.** Upset plot of GO annotations for genes indicated high methylation probability in water environment. High bars describe the number of genes engaged in common GO terms. The dots and lines merge GO terms with common genes. **E.** Upset plot of GO annotations for genes indicated high methylation probability in land environment. High bars describe the number of genes engaged in common GO terms. The dots and lines merge GO terms with common genes

Information on 2,190 probable aquatic methylation sites and 464 terrestrial methylation sites was revealed by analysis of raw Nanopore signals. Identifying 173 sites from 126 transcripts as significant in the water form (Additional file 2: Table S9) and 27 from 24 transcripts as significant in the land form (Additional file 2: Table S10) was based on the previously mentioned sites. The 16 methylation biases shared both forms (Fig. 3A and B). The CL.22551.1 transcript coded cytochrome-c oxidase/electron carrier was the most methylated transcript in the aquatic form, with five significant methylation sites. In the terrestrial form, the most frequently significantly methylated transcripts were CL.33843.1 encoded ribosomal protein S11 family protein, CL.6664.1 encoded papain family cysteine protease and CL.8794.1 translated 2-oxoglutarate (2OG) and Fe(II)-dependent oxygenase superfamily protein, each with two significant sites. Among the detected methylation sites in the aquatic form, CL.33844.1, encoded ribosomal protein S4 (RPS4A) family protein, exhibited the highest probability of methylation (0.97). Whereas, in the terrestrial form, CL.303.1 (Ribosomal protein S14p/S29e family protein) showed the highest methylation probability of approximately 0.9. Methylation was most frequently detected in the GAACT motif in both forms of *Riccia fluitans* (Fig. 3C). Transcripts with significant methylation sites in the aquatic form were involved in the following gene ontology processes (FDR < 0.05): aerobic (GO:0019646) and cellular respiration (GO:0045333) (Fig. 3D and Additional file 2: Table S11), while transcripts methylated frequently in the land form were involved in the chloroplast envelope (GO:0009941) and located within plastoglobules (GO:0010287) (Fig. 3E and Additional file 2: Table S12). An overlap was identified between aquatic methylation positions and unique DEGs identified by Illumina technology CL.28,438 (Gamma vacuolar processing enzyme), CL.28,820 (Low temperature and salt responsive protein family), CL.3354 (Disease resistance-responsive family protein), CL.19,054 (Peroxidase superfamily protein), and Nanopore technology CL.8117 (Chitinase family protein). Notably, the CL.2289 (Unknown) gene was shared between the methods. Similarly, terrestrial methylation positions showed overlap with Illumina DEGs and Nanopore DEGs. The unknown CL.3752 (Unknown) gene was identified as DEGs only in Nanopore sequencing technology. Other common elements, including genes CL.19,794 (Unknown), CL.21,493 (Unknown), CL.2593 (Mitochondrial import inner membrane translocase subunit Tim17/Tim22/Tim23 family protein), CL.31,915 (Carbonic anhydrase 2), were found to be relevant for both sequencing methods (Additional file 1: Fig. S6). Additional, transcript encoded Cytochrome P450 superfamily protein, which is DETs in short-read analysis, also revealed significant methylation modification in water environment (Additional file 1: Fig. S7). Three methylations of transcript CL.6664.1 were detected in aquatic *Riccia* and two other epitranscriptome events of the same transcript in the land form.

### Transition to aquatic environment results in longer poly(A) tails and different non-adenine modification patterns to mRNA transcripts

**Fig. 4:**
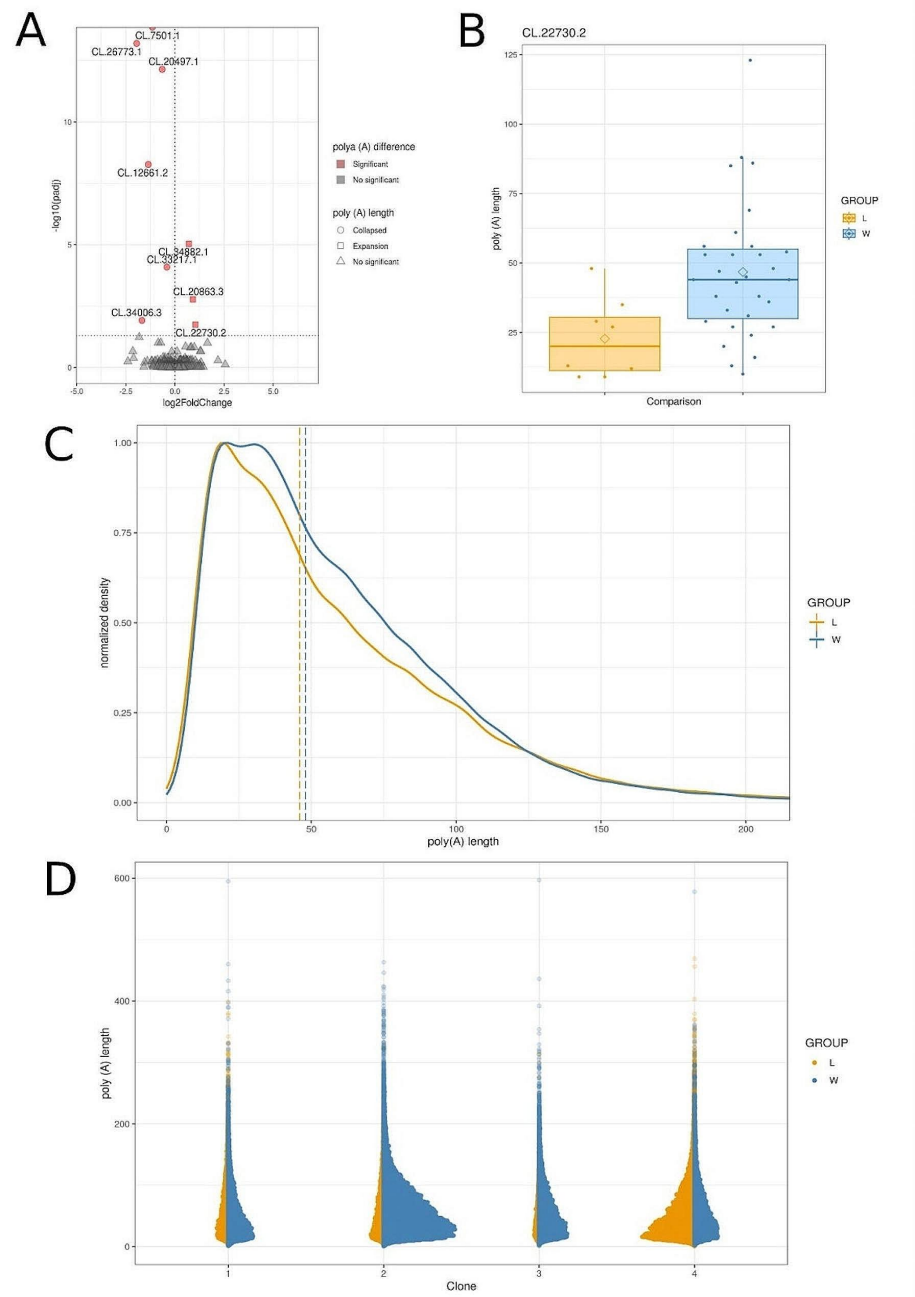
Polyadenylation signals detected by direct RNA sequencing. **A.** Volcano plot depicting log2 Fold Change (log2FC) for genes with significant poly (A) tail. The x-axis displays the log2FC values for each gene, while the y-axis shows the negative log-adjusted p-value (p-adjusted). The horizontal dashed line represents the negative logarithmic p-adjusted cutoff value (0.05), and the vertical line equals the value of 0 log2FC. Red points indicate statistically significant genes, while gray points represent non-significant genes. **B.** Boxplot comparing the distribution of poly(A) tail lengths in the CL.22730.2 transcript across study groups, water (blue) and land (orange). The boxplot depicts the median, first and third quartiles (lower and upper hinges), largest and smallest value (upper and lower whisker). **C.** Poly(A) tail length profiling of *Riccia fluitans* mRNA depending on the living environment. A density distribution plot is shown for the mRNA of all transcripts detected in *R. fluitans* cells in the aquatic environment (blue) and in the terrestrial environment (orange). The vertical dashed lines represent the median poly(A) tail length (in nucleotides). **D.** Scatter plot of poly(A) tail length for different *R. fluitans* clones, representing all transcripts from poly(A) tail length profiling. Each point represents the length of poly(A) from a clone grown in either an aquatic (blue) or terrestrial (land) environment

Deep transcriptomic direct RNA analysis revealed information on 156,906 polyA tails in *Riccia fluitans*, with 50,694 being identified in terrestrial and 106,212 in aquatic form of plants (Additional file 2: Table S14). Globally, the elongation bias of poly(A) tails was observed in the aquatic form of *R. fluitans* (p-value < 2.2e-16) (Fig. 4C and D). Nine transcripts exhibited significant differences in tail length, including CL.26773.1 (transcript coding - galactose oxidase/kelch repeat superfamily protein, CL.12661.2 (hydroxycinnamoyl-CoA shikimate/quinate hydroxycinnamoyl transferase), CL.34006.3 (Enoyl-CoA hydratase/isomerase family), CL.20497.1 (UDP-glucosyl transferase 73B1) and two unknown (CL.33217.1 and CL.7501.1) (Additional file 2: Table S15). The mentioned transcripts displayed elongated tails in their terrestrial environment, while CL.22730.2 (coding ABC-2 type transporter family protein), CL.20863.3 (serine carboxypeptidase-like 20), and CL.34882.1 (Rab5-interacting family protein) in the aquatic condition (Fig. 4A and B). The CL.22730.2 had the most tails isoform detected among statistically significant transcripts. In detail, 31 polyA tails were specific to aquatic form and 8 to the terrestrial variants (Fig. 4B).

**Fig. 5:**
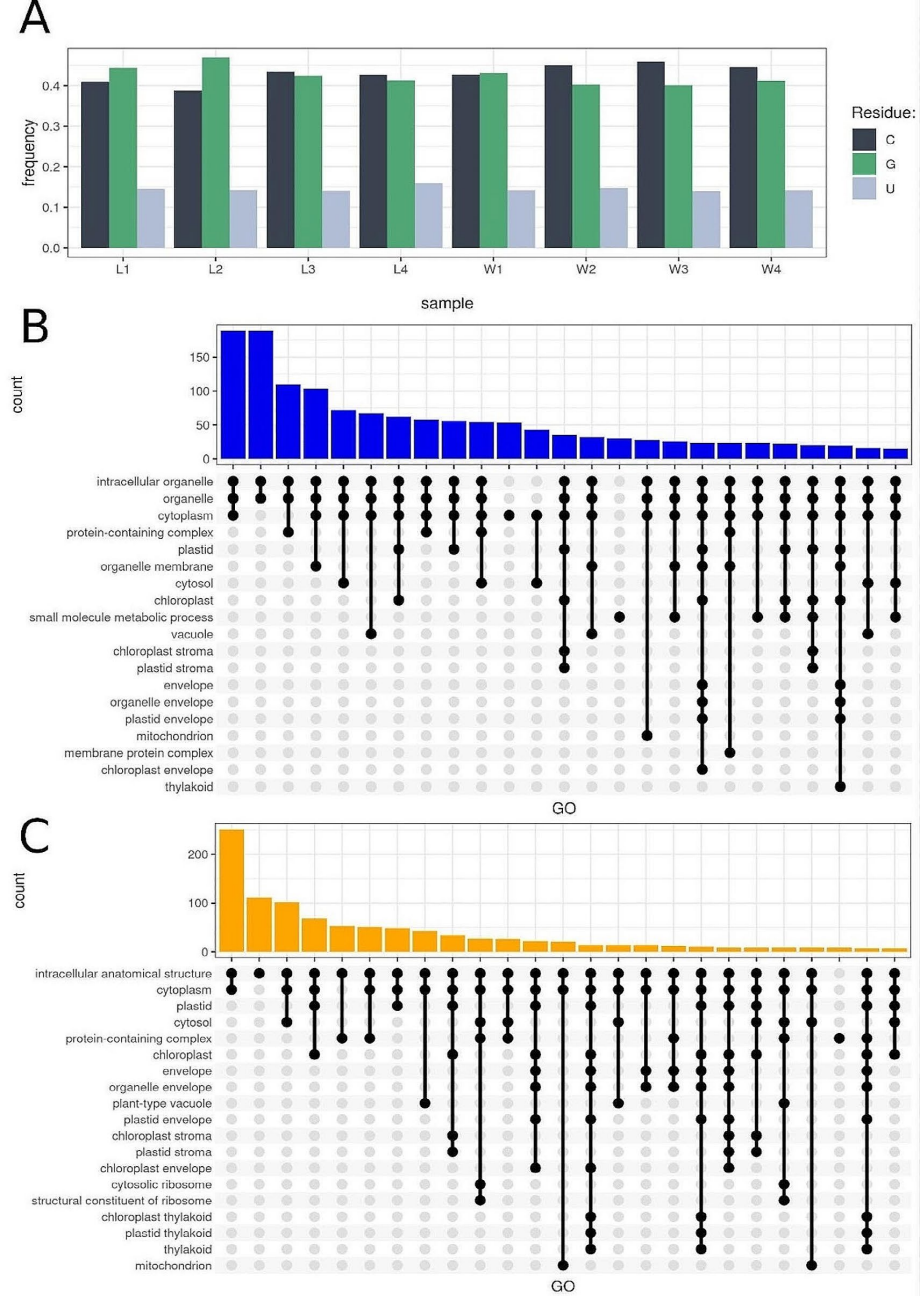
Non-adenine signals detected by direct RNA sequencing. **A.** Barplot depicts the frequency of non-adenine events in each sample. Dark green bars represent cytosine, light green bars represent guanine, and silver bars represent uracil. **B.** Upset plot of GO annotations for transcripts with non-adenine residues detected in the water environment. High bars describe the number of transcripts engaged in common GO terms. The dots and lines merge GO terms with common genes. **C.** Upset plot of GO annotations for transcripts with non-adenine residues in land environment. High bars describe the number of transcripts engaged in common GO terms. The dots and lines merge GO terms with common genes

We have shown that non-A modifications also occur in *Riccia fluitans.* 8,884 non-a observations were detected in water and 4,609 in land form. The most frequent non-A was cytosine with 3,979 observations in water-form *Riccia* and guanine with 1,976 observations in the land form (Fig. 5A). The unknown CL.7154.1 was marked as the most abundant non-A transcript in the aquatic environment, while the unknown CL.7156.1 in the land environment. Summarized number of non-A events in both environmentals, the highest number of non-A modifications were annotated in Cold, circadian rhythm, and rna binding 2 transcript (CL.11266.1) (Additional file 2: Table S16). Transcripts with non-a were involved in GO processes such as cytoplasm (GO:0005737), cytosol (GO:0005829), plastid (GO:0009536), organelle envelope (GO:0031967), and chloroplast (GO:0009507) (Fig. 5B and C and Additional file 2: Table S17 and S18).

## Discussion

Certain modifications like m6A, m5C, pseudouridine, and hm5U have been shown to increase error rates and reduce fidelity during reverse transcription into cDNA [20–22]. This is likely due to interference with proper Watson-Crick base pairing, causing misincorporations of incorrect nucleotides. RNA modifications can also cause premature termination or stalling of the reverse transcriptase enzyme upstream of the modification site [22, 23], leading to truncated cDNA products with reduced sequence coverage. Additionally, some modifications like pseudouridine may induce deletions or mutations in the synthesized cDNA sequence under certain conditions [23, 24], further reducing accuracy. These challenges posed by RNA modifications highlight the need for careful methodological considerations when conducting differential gene expression analysis. To address this, various analysis approaches can be utilized, each with its own advantages and limitations.Differential gene expression analysis, a fundamental and crucial transcriptomic analysis method, was conducted using two different approaches: short-read Illumina sequencing and direct RNA Nanopore sequencing. Moderate consistency was observed in this study, although this was lower than reported by Wongsurawat et al. (2022) [2], who used a Nanopore cDNA approach. This discrepancy is likely due to the use of long-read technology in the latter study, which may produce more consistent differential expression results. Factors contributing to this difference include sequencing depth and the underlying methods (PCR-based versus native RNA transcripts). Short-read technology provides the advantage of high sequencing depth. Additionally, PCR amplification allows detection of even lowly expressed genes, leading to their designation as differentially expressed genes (DEGs). As noted by Wu et al. (2023) [25], DEGs identified by both sequencing methods tend to have higher expression levels compared to those detected by only one method. Conversely, Nanopore direct RNA sequencing (even with enrichment) cannot generate the same level of sequencing depth, potentially hindering the detection of low-expressed genes. However, it avoids introducing PCR-related biases. Oxford Nanopore technology provides long reads, often encompassing full or near-full-length transcripts. Additionally, GC content is known to influence PCR amplification, potentially introducing bias in short-read Illumina sequencing candidate detection [26]. For our primary results, we prioritized Nanopore direct RNA sequencing, with short-read Illumina sequencing used solely for validation purposes. As this research represents the first investigation of *Riccia fluitans* water-land adaptation, we prioritize stringent and robust methods, particularly during the preparatory stages of our genomic investigations. Since no prior studies have explored plant expression profiles using both methods, we cannot definitively determine which approach is superior for liverwort transcriptome analysis. Furthermore, we believe that the combined use of both methods reduces concerns regarding potential statistical manipulation.

While RNA modifications can directly impact reverse transcription fidelity and coverage, gene expression analyses are still generally comparable between direct RNA sequencing and cDNA sequencing approaches [27]. The different pattern expression of transcript containing fasciclin-like domain revealed by cDNA and direct RNA analyses. These domains are essential for FASCICLIN-LIKE ARABINOGALACTAN PROTEINS (FLAs) function and are associated with cell adhesion functions [28–30]. Fasciclin domains are typically 110 to 150 amino acids long and contain two highly conserved regions, H1 and H2, of approximately 10 amino acids each. FLAs are widely distributed in plant tissues and play roles in plant growth, development, and stress response. In *Arabidopsis*, they have been found to impact secondary cell wall development, stem biomechanics, and cell wall architecture [28]. They are also involved in responses to stress and are thought to be involved in cell adhesion [28–31]. However, their function and structure in non-seed plants is poorly explored.

One of the main advantages of native RNA sequencing is ability detection of various transcripts modifications. N6-methyladenosine (m6A) is a prevalent and dynamic modification in eukaryotic RNA, playing a crucial role in various physiological aspects of living organisms, including growth, development, and stress responses [7]. The m6A modification is involved in the regulation of mRNA stability, alternative splicing, translation, export, and maturation of microRNA, which can influence the plant’s ability to adapt to environmental changes [32]. In plants, m6A RNA modification has been linked to abiotic stress responses, such as salt and osmotic stress, drought, cold and UV radiation [33–36]. For example, in *Arabidopsis thalliana* the m6A modification has been important for salt stress tolerance [35]. In the context of amphibious plants like analyzed *Riccia fluitans*, which exhibit remarkable adaptability to fluctuating aquatic and terrestrial environments, m6A RNA modification could potentially play a role in their fast adaptation to changing environments. One of the extensively methylated transcripts encode papain family cysteine proteases, which are involved in the response to abiotic stress. Zang et al. showed that transgenic *Arabidopsis* overexpressing the gene encoding a papain family cysteine protease exhibited stronger drought tolerance under water-stressed conditions than the wild type, suggesting that the gene plays a role in mediating dehydration tolerance [37]. The sweet potato papain family cysteine proteases 2 gene was involved in the response to darkness. In addition, the same gene in *Arabidopsis* increased resistance to drought and salt stress [38]. On the other hand, overexpression of the sweet potato papain family cysteine proteases 3 gene in *Arabidopsis* conferred sensitivity to drought stress [39]. Despite differences in methylated sites between water and land forms, the expression on gene complexes involved in m6A methylation processes is similar, which can be explained by high abundance modified transcripts in mRNA.

Similarly to m6A, the length of the poly(A) tail is another critical regulatory factor in mRNA metabolism. This modification influences stability, export, and translation, offering an additional layer of dynamic control in the plant’s stress response mechanisms. The poly(A) tail is not a static, simple entity that merely denotes the 3’ end. Rather, the poly(A) tail should be viewed as a dynamic and variable part of the transcript [40]. Polyadenylation, characterized by the addition of poly(A) tails to mRNA molecules, is a critical post-transcriptional modification influencing mRNA stability, nuclear export, and translation efficiency [41, 42]. In plants, the regulation of poly(A) tail length plays a pivotal role in responding to various stress conditions, thereby facilitating adaptive responses that ensure survival in changing environments [43]. Poly(A) tails act as a dynamic regulatory mechanism that can be modulated in response to stress,. Research has demonstrated that alternative polyadenylation (APA) leading to the generation of mRNA isoforms with differing poly(A) tail lengths, is a novel strategy for the regulation of gene expression in response to stresses in plants [43–45]. APA contributes to the diversification of the transcriptome and proteome under stress conditions, enabling plants to fine-tune the expression of genes involved in stress responses [41, 42]. For instance, in *Arabidopsis thaliana*, the poly(A) tail length of specific mRNAs has been shown to vary in response to heat shock, suggesting that the modulation of poly(A) tail length is a mechanism through which plants respond to thermal stress by controlling the stability and translation of heat shock protein (HSP) mRNAs [45]. This modulation ensures the rapid accumulation of HSPs, crucial for protein folding and protection under heat stress. Furthermore, the study of full-length RNA molecules across different tissues has revealed tissue-specific and evolutionarily conserved regulation of poly(A) tail length, indicating that this mechanism is fundamental to plant development and stress responses [46]. Changes in polyA tail length can significantly impact also the ability to withstand water stress in *A. thaliana* [44]. The mRNAs with longer poly(A) tails are generally more stable and efficiently translated, leading to an increased accumulation of proteins essential for stress response [41, 42]. This adaptive strategy enhances the plant’s resilience to water stress by improving its water retention and stress signaling pathways, ultimately contributing to its survival under adverse environmental conditions. Further studies on the role of polyA tail length in environmental adaptations of early land plants could shed new light in the molecular processes behind terrestrialization. The influence of U and G non-A at the end of poly(A) tails on mRNA stability regulation has been demonstrated, where they can either inhibit or promote poly(A) tail degradation [47, 48]. In *A. thaliana*, non-adenine nucleotides have been found in the polyA tail, suggesting that more uniform poly(a) tails in poly(A)-binding proteins may increase translation efficiency [49]. It will be interesting to investigate the dynamics of poly(A) tails in this liverwort under environmental changes, as we see clear differences in tail lengths under environmental changes and a global change in the number and proportion of non-A mutations in poly(A) tails.

## Conclusions

The study of *Riccia fluitans* utilizing nanopore direct RNA sequencing has provided valuable insights into how the plant’s gene expression changes when it moves from land to water. The study of native mRNA sequences showed differences in the length of poly(A) tails, m6A modifications, and expression patterns, pointing to intricate regulatory processes in the adaptation of *R. fluitans* to changing environments. Transcripts with altered poly(A) tail lengths and m6A modifications suggest a refined post-transcriptional regulatory mechanism in response to environmental cues. The variation in poly(A) tail length, specifically in transcripts related to stress responses and metabolism, signifies a strategic modulation of mRNA stability and translation as an adaptation strategy. Similarly, the variability in m6A modifications, particularly in transcripts coding for ribosomal proteins and enzymes, indicates a sophisticated mechanism for adjusting mRNA processing, translation, and decay in response to different conditions. The differential expression analysis complements these findings by highlighting genes that are differentially regulated depending on the environment. The downregulation of certain genes in terrestrial conditions and upregulation in aquatic conditions reflects a robust transcriptional response to environmental stressors. This differential expression extends to coding RNAs, long non-coding RNAs, and other RNA types, indicating a broad regulatory network that encompasses various RNA molecules.

## Materials and methods

### In vitro cultures of *Riccia fluitans*

Plant material was obtained from an axenic in vitro culture of *Riccia fluitans* RF.1 from a previous experiment [50]. Based on literature data and previous experiments [15, 16, 50] the *R. fluitans* plants grew on the ½GB5 medium with 20 g · l-1 sucrose, 8 g · l-1 agar-agar and pH 6.0. The upper fragments of sterile plants of *R. fluitans* were used as secondary explants and were placed on the medium in the form of five small clumps separated by approx. 1–2 cm. Plants were grown in climate chambers at 24 °C under long-day conditions with a 16:8 photoperiod (16 h light; 8 h dark). After four weeks of plant growth, one part of the in vitro cultures was overlaid with sterile diH20, and the second part was maintained unchanged. The proper part of the experiment was set up in four replicates and conducted for two weeks.

### RNA extraction, library preparation and sequencing

Total RNA was extracted using RNA Plant Mini Spin (Qiagen) kit according to the manufacturer protocol. Adequate RNA quality and quantity of RNA samples were ensured by Tapestation (Agilent) analysis using High Sensitivity RNA screening tape kit and Qubit 4 fluorimeter using HS RNA Assay kit. The purified total RNA was used for sequencing library preparation. Long-read native RNA libraries were prepared from 50 ng of poly(A)-tailed mRNA per sample using Direct RNA Sequencing Kit SQK-RNA002 (Oxford Nanopore Technologies) according to the manufacturer’s protocol. To remove rRNA from total RNA, NEBNext® Poly(A) mRNA Magnetic Isolation Module (New England Biolabs) was used. In the first step of library preparation SuperScript III Reverse Transcriptase (Thermo Fisher Scientific) was used to synthesize the strand complementary to RNA and thus to prepare RNA-cDNA hybrid. In the next step sequencing adapters were attached using T4 DNA Ligase 2 M U/ml (New England Biolabs) together with NEBNext® Quick Ligation Reaction Buffer. The libraries were quantified with Qubit dsDNA HS Assay Kit (ThermoFisher) and sequenced using MinION MK1C portable device (ONT) and FLO-MIN 106 Flow Cells R.9.4.1 (ONT) prepared for sequencing with Flow Cell Priming Kit EXP-FLP002 (ONT). The raw reads were basecalled using Dorado 0.4.3 (ONT) using the rna002_70bps_hac@v3 model on the NVIDIA RTX4090 GPU. The raw reads were deposited in the ENA EMBL-EBI database at the following numbers PRJEB72691.

Total RNA for short read procedure was extracted using RNA Plant Mini Spin (Qiagen) kit according to the manufacturer protocol. Adequate RNA quality and quantity of RNA samples were ensured by Tapestation (Agilent) analysis and High Sensitivity kit. The purified total RNA was used for sequencing library preparation. Short-read RNA-seq libraries were prepared using Truseq RNA library with Ribo-Zero option and sequenced using Illumina NovaSeq 6000 platform at Macrogen Inc. (Seoul, Korea). The raw reads were deposited in the ENA EMBL-EBI database at the following numbers PRJEB72692.

### Expression profiling based on short-reads

Sequencing quality was assessed using FastQC software (http://www.bioinformatics.babraham.ac.uk/). After RNA-Seq, Illumina adaptors and poly-A segments were excised using the Trimmomatic tool v.0.39 [51]. Reads shorter than 120 nucleotides (nt) and with an average quality score (PHRED) on leading and trailing sites < 20 were removed from the dataset. Next, high-quality reads were mapped to the draft genome (preprint Mazdziarz et al., 2023) using the STAR v.2.7.11a tool and following parameters: --outFilterMultimapNmax 20 --outFilterMismatchNmax 999 --outFilterMismatchNoverLmax 0.04 --alignSJoverhangMin 8 --alignSJDBoverhangMin 1 --alignIntronMin 20 --alignIntronMax 1,000,000 --alignMatesGapMax 1,000,000. Obtained BAM files were used to create annotations using the stringtie v.2.2.1 software [52].The stringtie aggregated individual GTF files from each sample and merge them to final annotations. Splicing variants of individual genes were obtained using the genomic annotations (GTF file) and the count values for genes and transcripts were calculated by featureCounts v.2.0.6 with default parameters [53]. For transcript expression level, Salmon v.0.13.1 tool was implemented as a mapper (following parameters were used: --genecode –gcBias) [54]. The numeric values of expressed transcripts were estimated by the tximport v.1.30.0 package [55]. The statistical test (based on a negative binomial model) implemented in the DESeq2 v.1.42.0 R library was used to compare expression profiles of water and land transcripts [56]. The following cut-off values for significant differentially expressed genes (DEGs) and transcripts were set: logarithmic fold change (log2FoldChange) > 1 and adjusted p-value (padj) < 0.05.

### Expression profiling based on long-reads

The long-read digital MinION signals were converted from POD5 to the FAST5 format using the pod5-file-format program (https://github.com/nanoporetech/pod5-file-format). Next, the transcriptomic sequences were basecalled by Guppy v.6.0.0 (https://nanoporetech.com/support) with –recursive –trim_strategy none parameters. The FASTQ raw reads were quality-checked and passed to the mapping steps (as a reference *Riccia fluitans* genome), supported by minimap2 v.2.26 software with -ax splice parameter [57]. Similar to short-reads analysis, the gene expression profiles produced by the long-reads sequencing method were also estimated using stringtie, featureCounts and DESeq2 softwares. For transcript level expression quantification, the above proceed BAM files were used again by bambu v3.2.4 software to estimate the transcript count expression matrix for multiple samples [58]. The differentially expressed genes (DEGs) and differentially expressed transcripts (DETs) statistical significance was determined with the following parameters: padj < 0.05 and absolute log2FoldChange > 1. The results from both methods (short - and long-reads) were intersected and only common results were considered as final transcriptomic DEGs and DETs results. Additionally, the transcriptomic sequences were divided into coding and non-coding groups. Two potential coding prediction softwares, CPC2 v.1.0.1 [59] and PLEK v.1.2 [60], classified transcripts into separate groups. According to those classifications, significant genes were named differentially protein-coding genes and differentially long non-coding RNAs (DELs). If there were discrepancies in identification of coding potential between the two programs, those RNA were signed as OtherRNA. Relationships between DEGs, DELs and OtherRNA were estimated by co-expression analysis. Pairs of DEGs-DELs, DEGs-OtherRNA, and DELs-OtherRNA with similar transcriptomic profiles were characterized based on the Pearson correlation coefficient (*r* > 0.8 and *p* < 0.05). The results were visualised using the ggplot2 v.3.4.4 [61] and circlize v.0.4.15 [62] R Bioconductor v.3.18 packages.

### Differential adenylation and non-adenine residue analysis

The FASTQ files were remapped with default –ax map-ont flags to the *Riccia fluitans* transcriptome, which was created by compilation of stringtie and gffread v.0.12.7 script [63]. The nanopolish v.0.14.1 program (https://github.com/jts/nanopolish) was used to extract tail information for each transcript. A Mann-Whitney U-test was used to compare the significance of differences in poly(A) tail lengths between environments. Finally, the nanotail v.0.1.0 package (https://github.com/smaegol/nanotail) was applied to run a statistical method based on the general linear model (glm) to determine the significance of any differences in tail length. Transcripts with an adjusted p-value < 0.05 were considered as statistically significant. Previously generated nanopolish outputs, sequencing summary generated by the Guppy bascaller, and fast5 files were used to identify non-adenine (non-A) sites in the poly(A) tail by the ninetails v.1.0.0 program (https://github.com/LRB-IIMCB/ninetails).

### Methylation profiling analysis

The long-reads and transcriptome mapping results were indexed with Samtools 1.7.2 (https://github.com/samtools/samtools). Nanopolish eventalign was designed to work on FAST5 files, which rely on the HDF5 library, hindering efficient parallel analysis. To address this, FAST5 files from the previous step were converted to BLOW5 using slow5tools v.1.0.0 [64] F5c v.1.1 [65] supports BLOW5 and enables the use of Nanopolish modules for indexing and eventalign. The m6A identification was performed with m6anet v.2.0.1 [66], with the -num_iterations 1000 flag. Results selected for further analysis - comprising the probability of modification at each position for each transcript - were thresholded at 0.6.

### Functional annotations of DEGs, DETs, methylation, polyadenylation and non-adenine profiles

All DEGs, DETs, transcript with significant polyA tail difference and methylation profile changes were annotated by blastp v.2.12.0 [67]. Due to a lot of *Marchantia polymorpha* gene symbol annotations are incomplete and uncharacterized in databases, the identification process of *Riccia fluitans* translated genes/transcripts was based on *Arabidopsis thaliana* protein sequences. For blastp homology searching an e-value < 10e-5 was set as the cut-off threshold. This comparison facilitated the acquisition of descriptions and symbols for newly annotated *Riccia* proteins. The resulting gene signatures, DEG, DET and other epitranscriptome candidates were subsequently scanned for enrichment in Gene Ontology (GO) function annotations using g: Profiler v.0.2.2 R library with gost function [68]. Biological processes (BP), cellular components (CC), and molecular functions (MF) were annotated as ontological terms for the essential genes. Enrichment analysis with a false discovery rate (FDR) cut-off < 0.05 was employed to identify GO and pathway annotations regulated by differentially genes. The functional connection between DEG, DET and other epitranscriptome modifications of *Riccia fluitans* were visualized by highlighting those events using the ggplot2 R package.

### Electronic supplementary material

Below is the link to the electronic supplementary material.

Supplementary Material 1

Supplementary Material 2

## Data Availability

The raw reads were deposited in the ENA EMBL-EBI database at the following numbers PRJEB72691 and PRJEB72692. The genome and gtf file were deposited in the figshare database at the following link: https://figshare.com/projects/Riccia_fluitans/201057.

## Abbreviations

APA: Alternative Polyadenylation
BP: Biological processes
CC: Cellular components
DEG: Differentially expressed gene
DET: Differentially expressed transcript
DEL: Differentially expressed long non–coding RNA
FDR: False Discovery Rate
FLA: Fascilin–like arabinogalactan protein
GO: Gene ontology
hm5A: 5–hydroxymethyluridine
HSP: Heat shock protein
m6A: N6–methyladenosine
m5C: 5–methylcytosine
MF: Molecular functions
mRNA: messenger RNA
polyA: polyadenylation
SBS: Sequencing by synthesis
