# Supplementary material for: Epitranscriptome insights into Riccia fluitans L. (Marchantiophyta) aquatic transition using nanopore direct RNA sequencing
Source: BMC Plant Biol. 2024 May 15;24:399. doi: 10.1186/s12870-024-05114-4 (PMC11094948; doi:10.1186/s12870-024-05114-4)
Supplement: Supplementary file 1 — Supplementary Material 1 [file 12870_2024_5114_MOESM1_ESM.docx]

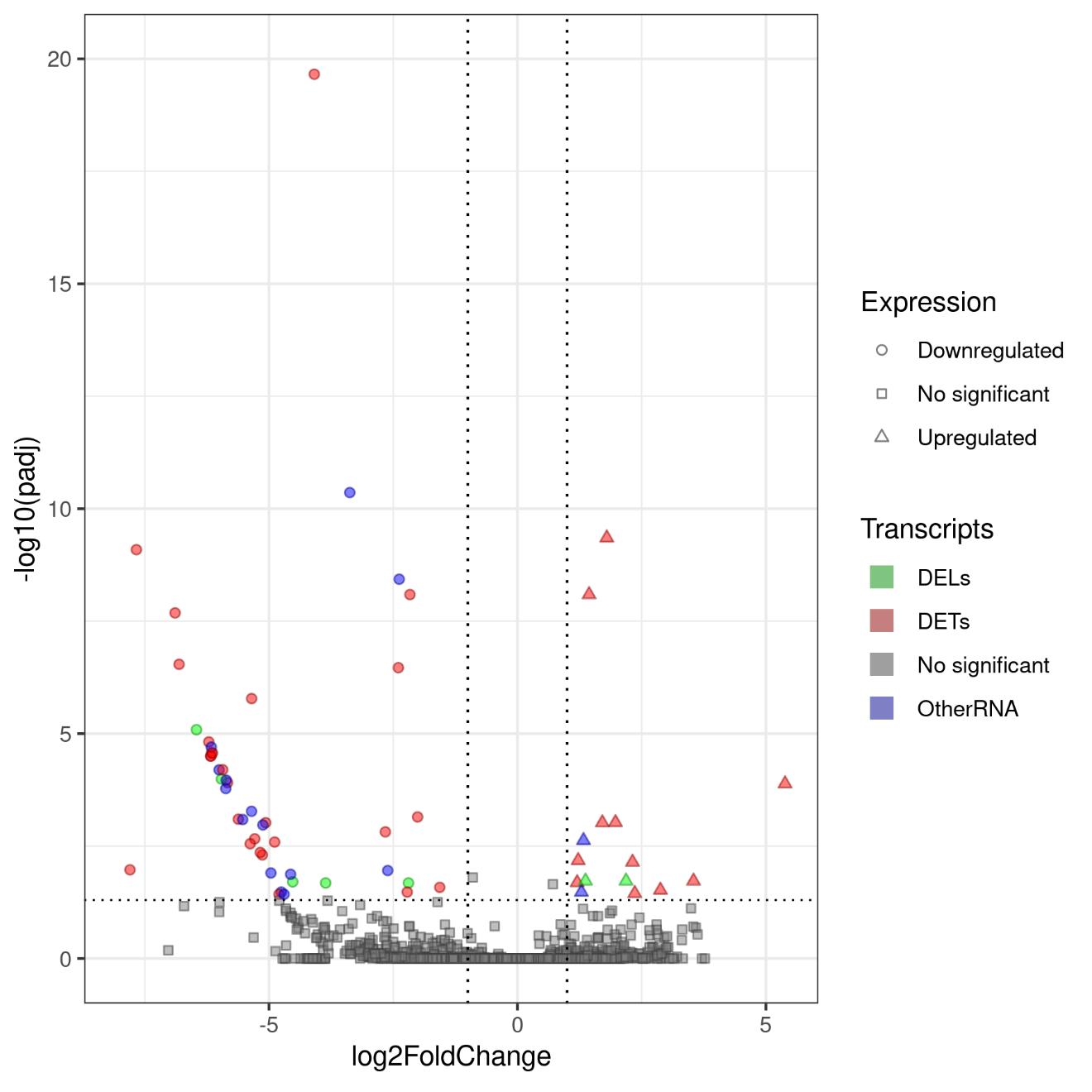


**Figure. S1.** Volcano plot depicting log2 Fold Change (log2FC) for significant transcripts detected in the Nanopore data. The x-axis displays the log2FC values for each transcript, while the y-axis shows the negative log-adjusted p-value (p-adjusted). The horizontal dashed line represents the negative logarithmic p-adjusted cutoff value (0.05) and the two vertical lines show the absolute value of log2FC equal to 1. The colored points indicate statistically significant transcripts, while grey points represent non-significant genes.


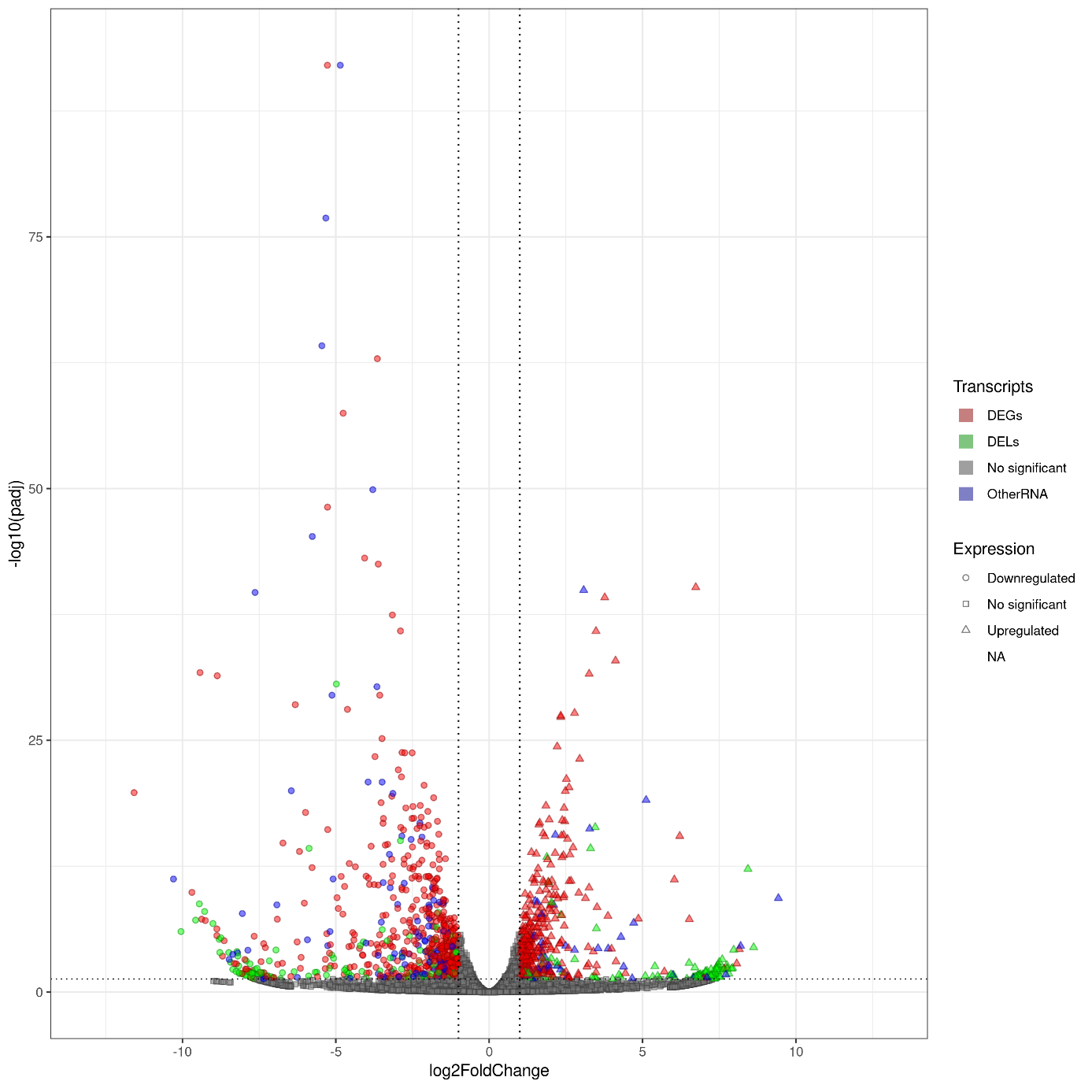


**Figure. S2.** Volcano plot depicting log2 Fold Change (log2FC) for significant genes detected in the Illumina data. The x-axis displays the log2FC values for each gene, while the y-axis shows the negative log-adjusted p-value (p-adjusted). The horizontal dashed line represents the negative logarithmic p-adjusted cutoff value (0.05), and the two vertical lines depict the absolute value of log2FC equal to 1. Colored points indicate statistically significant genes, while gray points represent non-significant genes.


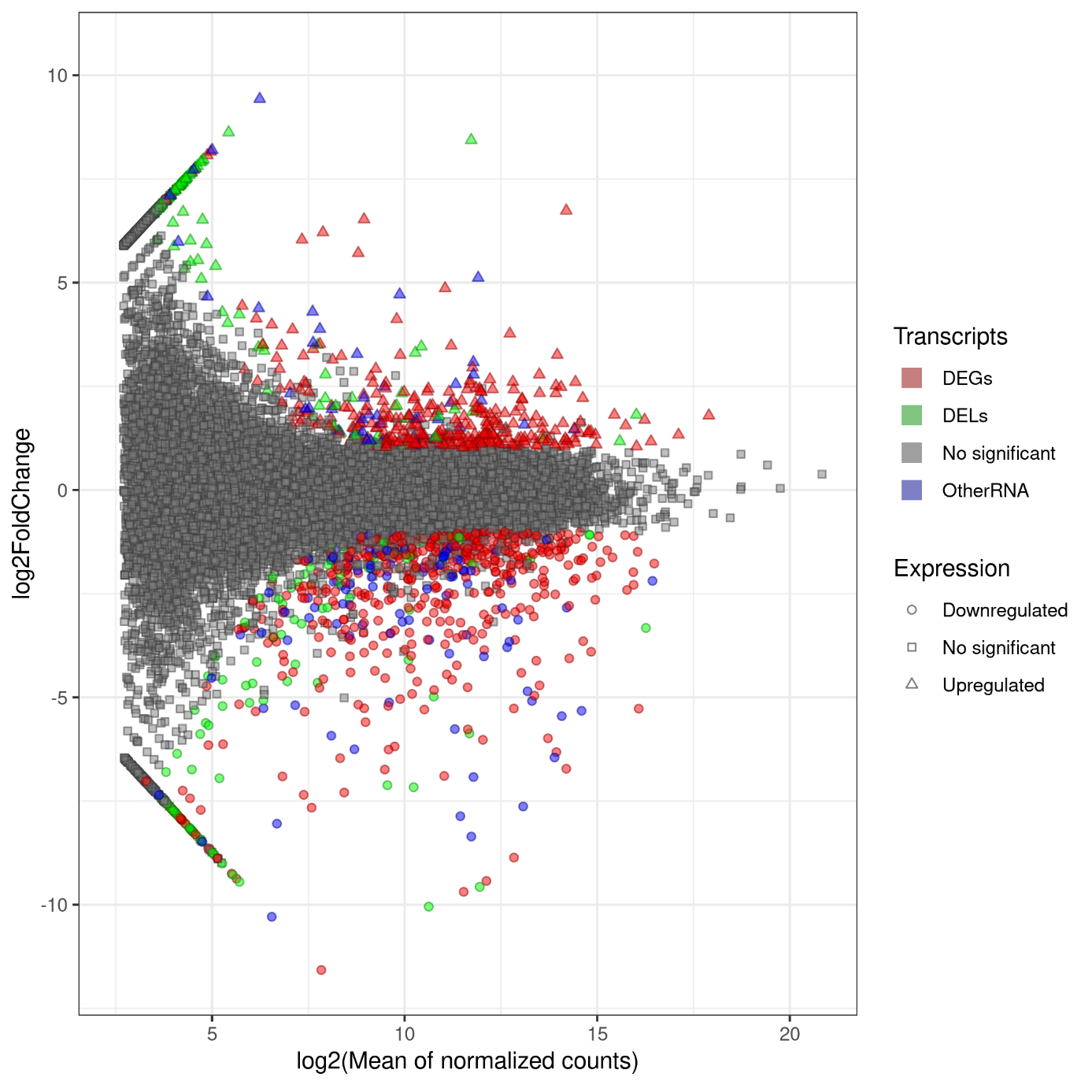


**Figure. S3.** The MA plot visualizes the association between log2FoldChange and log2 from the average of normalized counts for genes from Illumina data. The x-axis displays the log2 of the average of the normalized transcript counts, while the y-axis illustrates the log2FoldChange for the transcript. Squares represent irrelevant transcripts, triangles represent upregulated transcripts, circles represent downregulated transcripts, and the green colour indicates DELs, red indicates DEGs, blue indicates other RNAs, and grey indicates no significant transcripts.


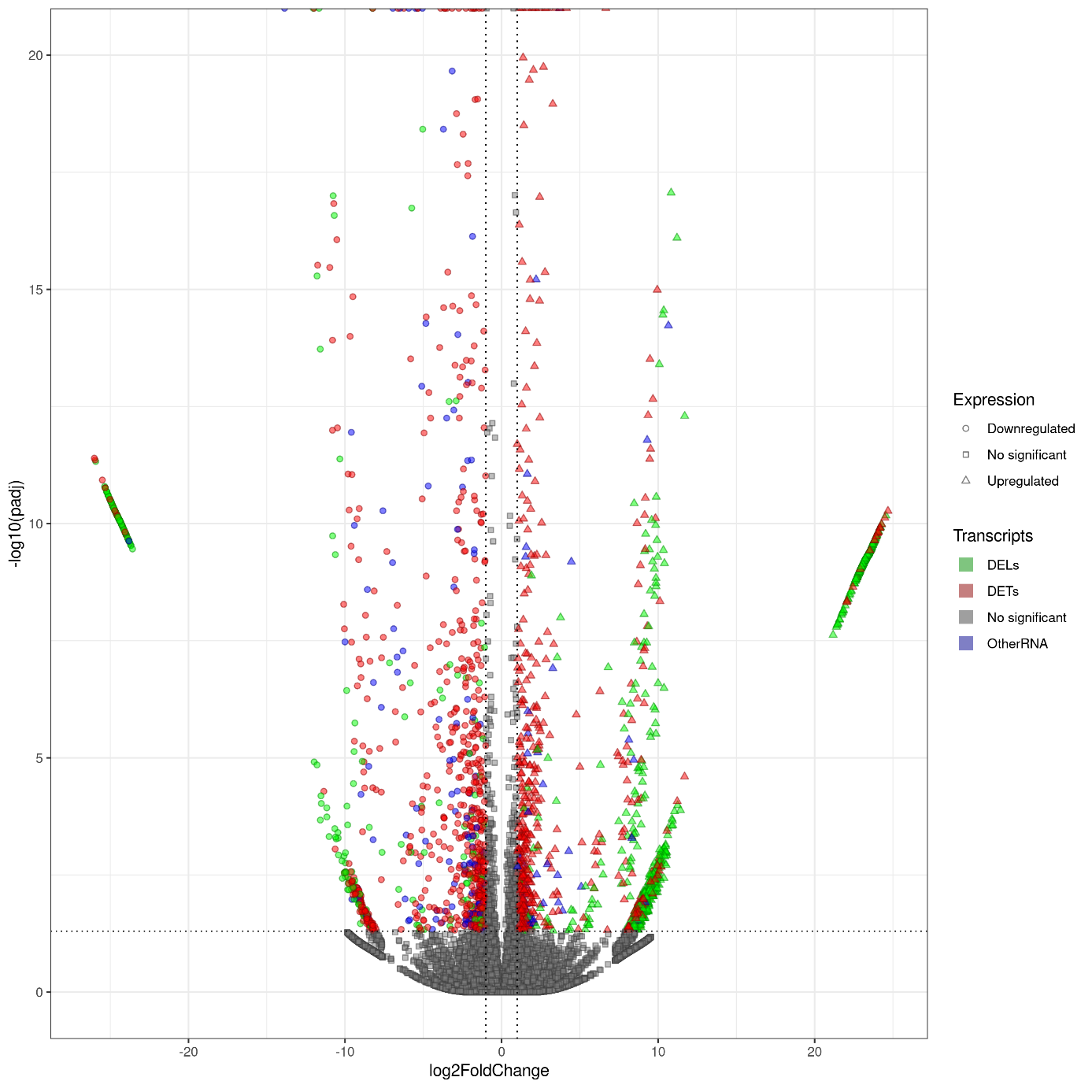


**Figure. S4**. Volcano plot depicting log2 Fold Change (log2FC) for significant transcripts detected in the Illumina data. The x-axis displays the log2FC values for each transcript, while the y-axis shows the negative log-adjusted p-value (p-adjusted). The horizontal dashed line represents the negative logarithmic p-adjusted cutoff value (0.05), and the two vertical lines show the absolute value of log2FC cut-off (1). Colored points indicate statistically significant transcripts, while grey points represent non-significant genes.


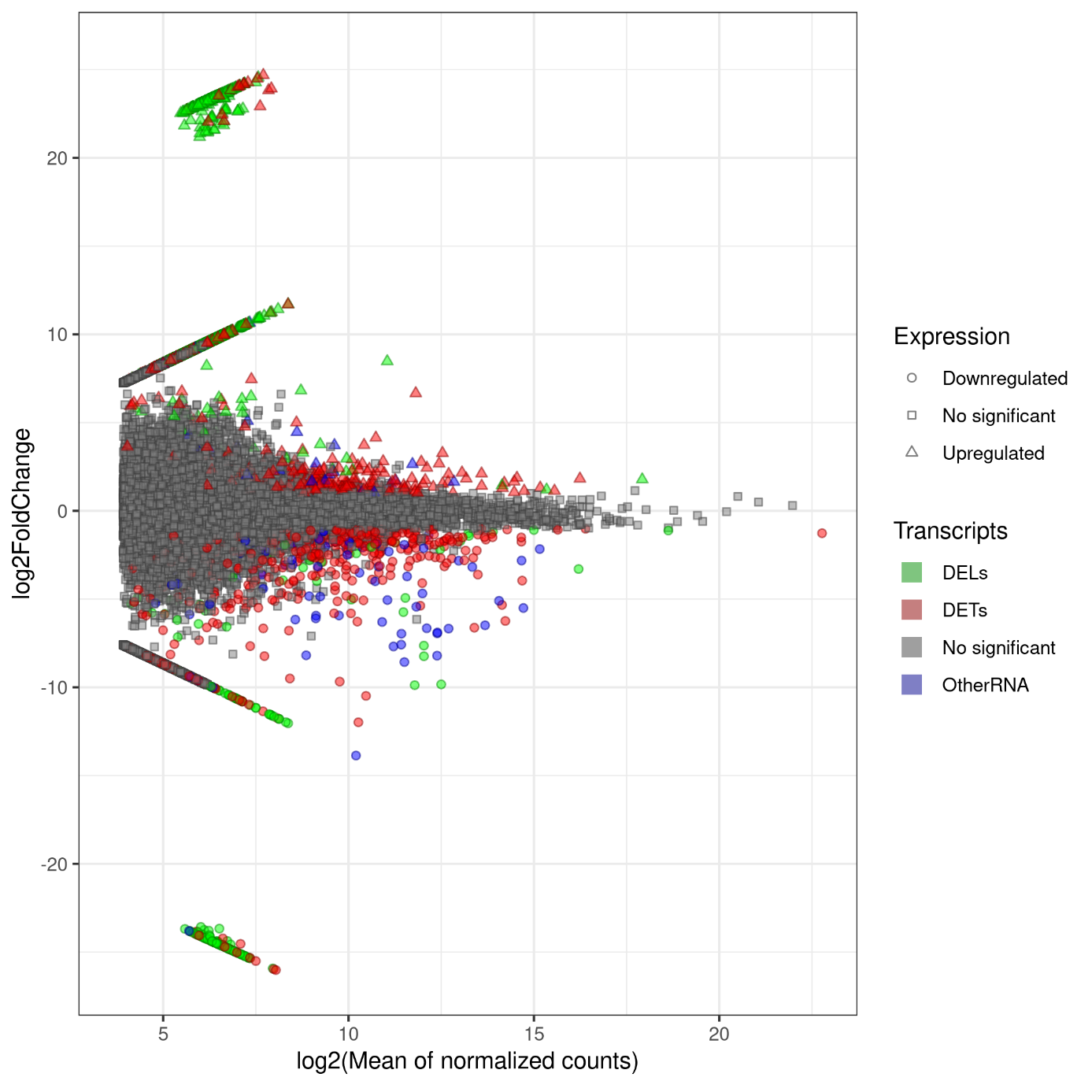


**Figure. S5.** The MA plot visualizes the association between log2FoldChange (x-axis) and average of normalized counts (y-axis) for transcripts detected by Illumina method. Squares represent irrelevant transcripts, triangles represent upregulated transcripts, circles represent downregulated transcripts, and the green colour indicates DELs, red indicates DETs, blue indicates OtherRNA, and grey indicates no significant transcripts.


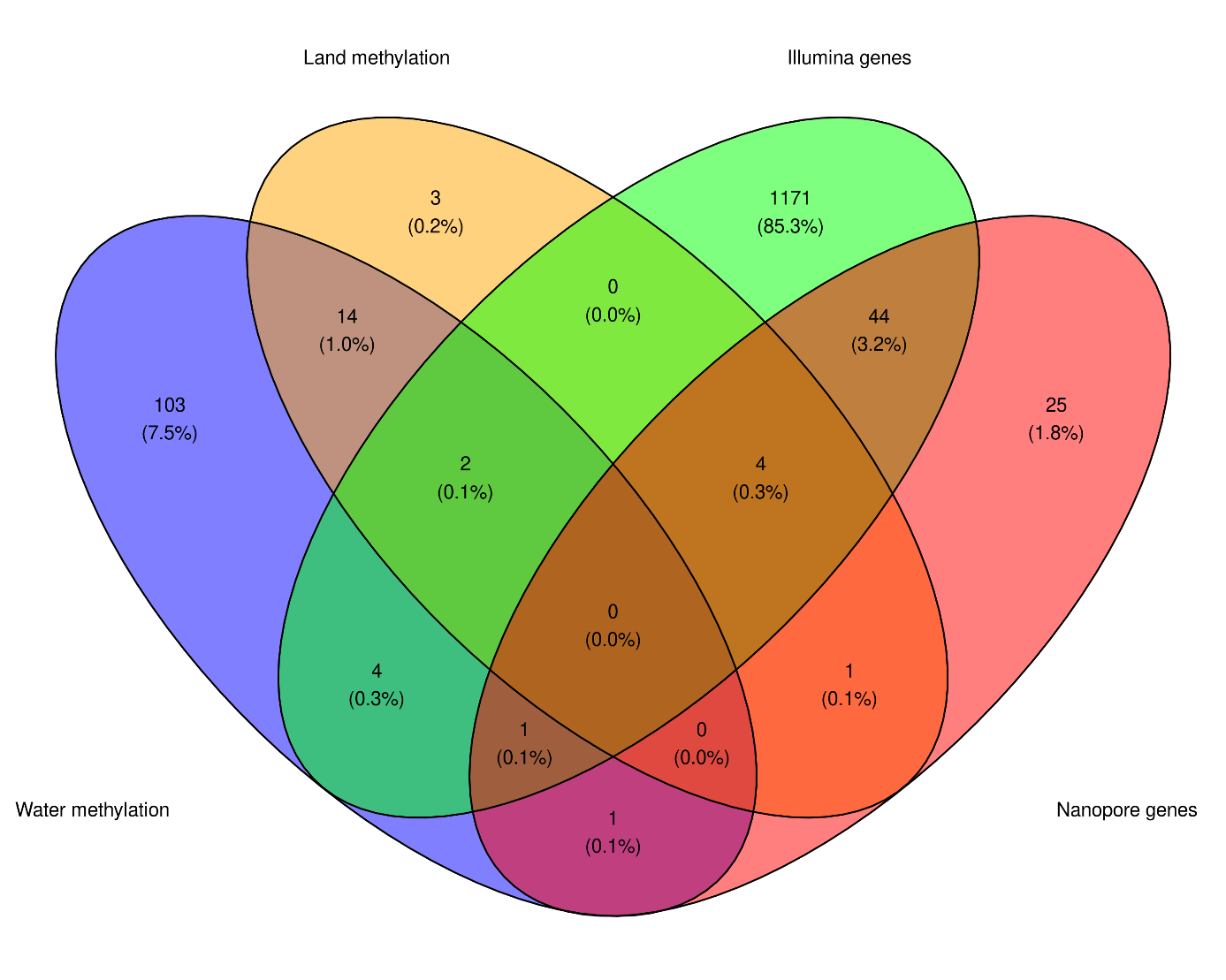


**Figure. S6.** Venn diagram represents the number, percentage, and intersecting parts of significantly expressed genes detected in Illumina (green), Nanopore (red), and genes that are significantly methylated in the water form (blue) and land form (orange) of *Riccia fluitans*.


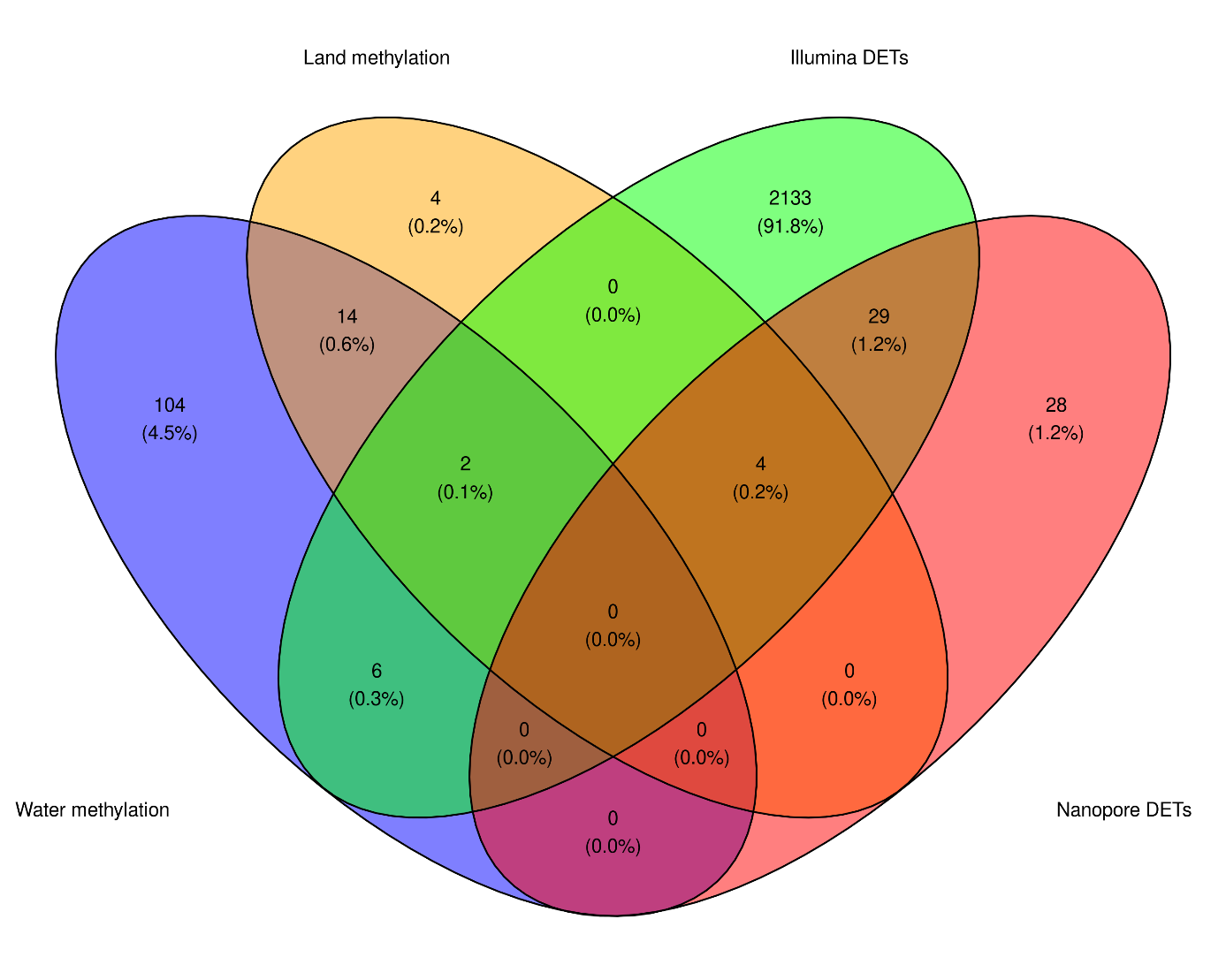


**Figure. S7.** Venn diagram represents the amount, percentage, and intersecting parts of significantly expressed transcripts detected in Illumina (green), Nanopore (red), and genes that are significantly methylated in the water form (blue) and land form (orange) of *Riccia fluitans*.
